# Therapeutic Antibodies in Hematology: Advances in Malignant and Non-Malignant Disorders

**DOI:** 10.3390/cells15010046

**Published:** 2025-12-25

**Authors:** Hiroshi Yasui, Masashi Idogawa, Tadao Ishida, Kohzoh Imai

**Affiliations:** 1 Department of Hematology and Oncology, St. Marianna University School of Medicine, Kawasaki 216-8511, Japan; 2 The Institute of Medical Science, The University of Tokyo, Tokyo 108-8639, Japan; 3 Division of Medical Genome Sciences, Department of Genomic and Preventive Medicine, School of Medicine, Sapporo Medical University, Sapporo 060-8556, Japan; idogawa@sapmed.ac.jp; 4 Department of Hematology, Japanese Red Cross Medical Center, Tokyo 150-8935, Japan; i.s.h.i.28@rondo.ocn.ne.jp; 5 Sapporo Medical University, Sapporo 060-8556, Japan; 6 Institute for Genetic Medicine, Hokkaido University, Sapporo 060-0808, Japan; 7 Kanagawa Cancer Center Research Institute, Yokohama 241-8515, Japan

**Keywords:** therapeutic antibody, hematology, antibody engineering, antibody–drug conjugates, bispecific antibodies

## Abstract

**Highlights:**

**What are the main findings?**

- Therapeutic antibodies, including monoclonal antibodies, antibody–drug conjugates, and bispecific antibodies, have fundamentally transformed the treatment of both malignant and non-malignant hematologic disorders.
- Advances in antibody engineering, such as T-cell–redirecting bispecific antibodies and structure-guided optimization, have expanded therapeutic efficacy but also revealed new challenges, including treatment-related toxicity and antigen escape.

**What is the implication of the main finding?**

- Future antibody-based therapies in hematology will require integrated strategies that balance efficacy and safety by addressing cytokine-related toxicities, off-target effects, and resistance mechanisms.
- Rational multi-antigen targeting, supported by structural prediction and molecular profiling, is expected to enhance the durability and precision of next-generation immunotherapies.

**Abstract:**

Therapeutic antibodies have revolutionized hematology, offering targeted and effective treatments for both malignant and non-malignant diseases. In hematologic malignancies, anti-CD20, anti-CD19, anti-CD38, and anti–B-cell maturation antigen (BCMA) antibodies have markedly improved survival outcomes, whereas antibody–drug conjugates and bispecific antibodies continue to expand therapeutic possibilities. Besides cancer, complement inhibitors such as eculizumab, ravulizumab, and the recently approved crovalimab have redefined paroxysmal nocturnal hemoglobinuria and atypical hemolytic uremic syndrome management, and the bispecific antibody emicizumab has transformed prophylaxis in hemophilia A. Furthermore, novel antibody formats such as the trifunctional anti-CD38 × CD3 antibody (Tri-31C2) exhibit enhanced anti-myeloma activity compared to chimeric CD38 antibodies, underscoring the future potential of T-cell–redirecting designs. This review summarizes key developments in therapeutic antibodies for hematological disorders, their action mechanisms, and emerging strategies to further optimize their efficacy and safety.

## 1. Introduction

Monoclonal antibodies have fundamentally reshaped hematology by enabling the selective targeting of disease-driving cells, signaling pathways, and immune microenvironment components. Since the approval of rituximab in 1997, antibody-based therapy has become integral to the management of both malignant and non-malignant hematologic disorders, ranging from B-cell lymphoma and multiple myeloma to paroxysmal nocturnal hemoglobinuria, hemophilia A, and immune cytopenia.

Continuous antibody engineering advances, including Fc modification, antibody–drug conjugates (ADCs), bispecific antibodies, and pH-dependent recycling technologies, have expanded the potency and versatility of antibody therapeutics. These platforms now encompass direct cytotoxic antibodies, payload-delivering ADCs, and T-cell–redirecting bispecific constructs, each with distinct immunologic and mechanistic advantages.

**Figure 1:**
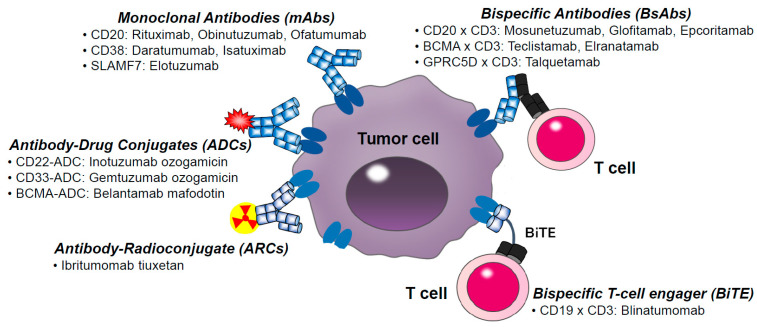
Therapeutic Antibody Classes Used in Hematological Malignancies. This schematic illustrates the principal classes of therapeutic antibodies used to treat haematological malignancies. (1) Monoclonal antibodies (e.g., anti-CD20 or anti-CD38) bind to surface antigens on malignant cells and exert antitumor effects through ADCC, CDC, antibody-dependent cellular phagocytosis, and signaling inhibition. (2) Antibody-drug conjugates (ADCs) and radioimmunoconjugates deliver cytotoxic or radioactive payloads to tumor cells upon antigen binding, enabling targeted intracellular killing. (3) Bispecific antibodies engage CD3 on T cells and tumor-associated antigens on malignant cells, redirecting cytotoxic T lymphocytes and inducing potent serial tumor cell lysis. These platforms collectively represent the core immunotherapeutic strategies that reshape the management of B-cell lymphomas, B-ALL, and multiple myeloma.

This review summarizes recent progress in therapeutic antibodies for hematologic malignancies and immune-mediated disorders and highlights emerging principles in molecular engineering, structural prediction, and clinical translation. A schematic overview of the major therapeutic antibody classes used in hematology is displayed in Figure 1.

## 2. Antibody Therapeutics in Hematologic Malignancies

Therapeutic antibodies are central components of modern treatment strategies for hematological malignancies. A broad antibody modality range, including monoclonal antibodies, ADCs, radioimmunoconjugates, and T-cell-redirecting bispecific antibodies, has received regulatory approval in the United States, Europe, and Japan for the treatment of B-cell lymphomas, B-cell acute lymphoblastic leukemia (B-ALL), and multiple myeloma. Table 1 provides an overview of approved antibody therapeutics and their primary indications.

### 2.1. B-Cell Malignancies

Antibody therapeutics have transformed the management of B-cell malignancies, beginning with anti-CD20 monoclonal antibodies and subsequently expanding to include radioimmunoconjugates, next-generation glycoengineered antibodies, and T-cell–redirecting bispecific platforms. Notably, these advances have both improved B-cell lymphoma outcomes and revolutionized B-ALL treatment through CD19-directed T-cell engagement. The following subsections summarize the key developments in CD20-based therapy, emerging CD20 × CD3 bispecific antibodies, and CD19 × CD3 bispecific antibodies for B-ALL.

#### 2.1.1. Anti-CD20 Antibody Evolution

Rituximab marked the beginning of antibody-based treatment for B-cell lymphomas. Nonetheless, intrinsic and acquired resistance, including low CD20 expression, complement resistance, and effector cell exhaustion, have led to the development of next-generation agents. Clinical use of rituximab has revealed resistance mechanisms including trogocytosis-mediated antigen loss, complement exhaustion, and attenuation of Fc-dependent effector functions. These phenomena are increasingly recognized as class effects that may influence the durability of responses to therapeutic monoclonal antibodies. Obinutuzumab, a glycoengineered type II anti-CD20 antibody, was designed to enhance antibody-dependent cellular cytotoxicity (ADCC) through afucosylated Fc regions and induce direct non-apoptotic cell death via actin cytoskeleton remodeling [1]. In the GALLIUM trial, obinutuzumab-based immunochemotherapy demonstrated improved progression-free survival compared to rituximab-based immunochemotherapy in follicular lymphoma, leading to its approval across major regulatory agencies. Ofatumumab, a fully human anti-CD20 antibody, targets a membrane-proximal epitope distinct from that of rituximab and exhibits strong complement-dependent cytotoxicity (CDC). Although its use has declined following the advent of obinutuzumab and targeted therapies for CLL, ofatumumab has provided an important proof-of-concept for epitope engineering in CD20 therapy [2]. Ibritumomab tiuxetan, a radiolabeled anti-CD20 antibody (90Y-ibritumomab), is an innovative strategy that combines targeted antibody delivery with radioisotopes. Despite its reduced use in the current era, ibritumomab has demonstrated durable responses in indolent B-cell lymphomas and remains an important milestone in antibody–radioconjugate development [3].

#### 2.1.2. CD20 × CD3 Bispecific Antibodies

The emergence of CD20 × CD3 bispecific antibodies has transformed the treatment paradigms for relapsed or refractory B-cell lymphomas by enabling T-cell engagement independent of native antigen presentation. A pivotal preclinical study by Sun et al. demonstrated that full-length humanized CD20 × CD3 bispecific IgG (CD20-TDB) induces potent T-cell–dependent cytotoxicity against CD20-positive malignant B cells across multiple models [4]. This molecule strictly activates both CD4+ and CD8+ T cells in the presence of CD20-expressing targets, mediates perforin/granzyme-dependent killing, and remains effective even when CD20 expression is extremely low or when competing anti-CD20 antibodies, such as rituximab, are present. Notably, CD20-TDB achieved near-complete B cell depletion in humanized and double-transgenic mouse models and showed full activity in cynomolgus monkeys while maintaining IgG-like pharmacokinetics. These mechanistic and translational insights provide a key foundation for the subsequent development of clinically approved CD20 × CD3 bispecific antibodies (BsAbs).

Mosunetuzumab, the first approved CD20 × CD3 bispecific antibody, induces serial T-cell–mediated cytotoxicity and has demonstrated high complete response rates in patients with heavily pretreated follicular lymphoma, including those refractory to anti-CD20 antibodies [5,6]. Glofitamab, characterized by a 2:1 CD20:CD3 binding configuration, delivers a highly potent cytotoxic synapse and enables a time-limited treatment course. In relapsed/refractory DLBCL, glofitamab achieves durable remission even after CAR-T failure [7]. Epcoritamab, a subcutaneously administered CD20 × CD3 antibody, offers comparable efficacy with a more favorable administration profile and reduced cytokine-release-related toxicity, supporting its use in outpatient settings for relapsed/refractory DLBCL [8] and relapsed/refractory follicular lymphoma [9]. Collectively, these bispecific antibodies represent a new pillar of B-cell lymphoma therapy, complementing CAR-T cells and providing accessible off-the-shelf treatment options for refractory diseases.

#### 2.1.3. Antibody-Based Therapies in B-ALL

Antibody-based immunotherapy has profoundly transformed the treatment paradigm of B-ALL. Two major therapeutic platforms, CD19 × CD3 bispecific T-cell engagers and CD22-directed ADCs, have significantly improved the outcomes in relapsed/refractory (R/R) and minimal residual disease (MRD).

##### CD19 × CD3 Bispecific Antibody

Blinatumomab, a CD19 × CD3 bispecific T-cell engager (BiTE), activates cytotoxic T cells independently of major histocompatibility complex (MHC) restriction and induces serial killing of CD19-positive leukemic blasts. Clinical studies have demonstrated its transformative impact on multiple diseases. In relapsed/refractory B-ALL, blinatumomab significantly improved overall survival compared with conventional chemotherapy and achieved high complete remission rates, even in heavily pretreated patients [10]. In MRD-positive B-ALL, blinatumomab induces deep molecular remission, resulting in high rates of MRD negativity and prolonged relapse-free survival [11]. Emerging evidence indicates that the benefits of blinatumomab extend beyond those for MRD-positive diseases. A recent prospective study in adults with MRD-negative B-ALL demonstrated that consolidation therapy with blinatumomab was safe, reduced the risk of relapse, and yielded excellent long-term survival outcomes [12]. Notably, the treatment was well tolerated, with low rates of severe cytokine release syndrome or neurotoxicity, underscoring its potential role as a post-remission strategy to deepen and stabilize molecular remission, even in patients who have already achieved MRD negativity.

##### CD22-Directed Antibody–Drug Conjugate

Inotuzumab ozogamicin (CMC-544) is a humanized anti-CD22 monoclonal antibody that is conjugated to calicheamicin, a highly potent cytotoxic antibiotic. After binding to CD22, the antibody-drug conjugate is rapidly internalized, leading to the intracellular release of calicheamicin. Calicheamicin binds to the minor groove of DNA, inducing double-strand breaks and subsequent apoptosis [13]. In the pivotal INO-VATE ALL trial for patients with relapsed/refractory B-ALL, inotuzumab ozogamicin demonstrated markedly superior efficacy compared to standard chemotherapy, achieving significantly higher complete remission rates (80.7% vs. 29.4%) and deeper molecular responses, including higher rates of MRD negativity. Veno-occlusive liver disease is the principal treatment-related adverse event associated with inotuzumab ozogamicin [14].

### 2.2. Antibody Therapeutics in Multiple Myeloma

The treatment landscape for multiple myeloma (MM) has dramatically evolved with the introduction of monoclonal antibodies targeting plasma cell–specific surface antigens. Foundational reviews, such as those by Yasui et al., emphasize the central role of the immune microenvironment in MM and anticipate the development of immune-based therapeutic approaches [15]. These early insights have helped frame the subsequent evolution of antibody-directed and T-cell–redirecting therapies that are now central to MM management. In addition to proteasome inhibitors and immunomodulatory drugs, antibody-based therapies have also significantly improved survival outcomes. Currently, clinically approved or investigational antibodies for MM include those targeting CD38, SLAMF7, and BCMA, as well as next-generation bispecific constructs and ADCs.

#### 2.2.1. Anti-CD38 Antibodies

CD38 is a transmembrane glycoprotein that is highly expressed in plasma cells and functions as both a receptor and an ectoenzyme. Daratumumab, the first anti-CD38 antibody approved in 2015, mediates ADCC, CDC, and antibody-dependent cellular phagocytosis. In addition to immune effector–dependent killing, isatuximab, another anti-CD38 antibody with a distinct epitope and mechanism, can induce direct cytotoxicity. Our recent mechanistic studies have demonstrated that isatuximab triggers direct lysosome-mediated cell death, specifically in MM cells with chromosome 1q gain or amplification (1q+), characterized by FOXM1, NEK2, and UBE2T upregulation [16]. Moreover, isatuximab promotes internalization into CD38^high^ cells, leading to lysosomal enlargement and protease-mediated degradation of FOXM1, a transcription factor essential for the proliferation and redox homeostasis of 1q+ MM cells. This process induces reactive oxygen species (ROS) accumulation and non-apoptotic cell death independent of immune effector cells. Furthermore, combination therapy with pomalidomide or carfilzomib enhanced cytotoxicity by augmenting FOXM1 downregulation and ROS production. Therefore, the FOXM1–NEK2–UBE2T axis is a molecular vulnerability in 1q+ MM and provides a rationale for isatuximab-based regimens targeting high-risk cytogenetic subgroups.

Early mechanistic studies demonstrated that immunomodulatory drugs (IMiDs), such as lenalidomide, significantly enhance antibody-mediated cytotoxicity by activating natural killer (NK) cells and strengthening ADCC. Foundational studies by Hayashi et al. demonstrated that IMiDs upregulate granzyme B, perforin, and immune synapse formation, thereby potentiating the antitumor efficacy of therapeutic antibodies [17]. These insights provide a biological basis for clinical regimens such as daratumumab–lenalidomide for multiple myeloma and rituximab–lenalidomide (R^2^) for follicular lymphoma, which are now established standards of care.

**Figure 2:**
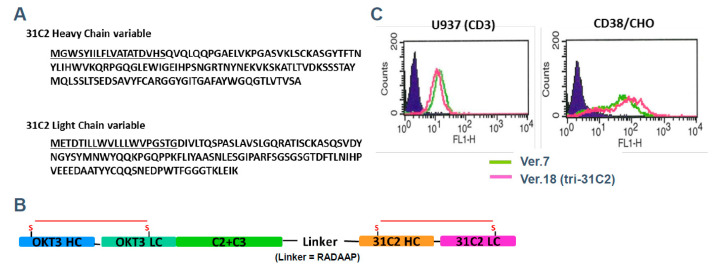
Structure and functional characterization of the tri-31C2 bispecific antibody. (**A**) Schematic representation of the tri-31C2 antibody, which combines the variable regions of the humanized anti-CD3 antibody OKT3 and the chimeric anti-CD38 antibody ch-31C2, fused to a human IgG1 Fc region. (**B**) Expression vector design for tri-31C2. The 31C2-HC and 31C2-LC domains were derived from the heavy- and light-chain variable regions of ch-31C2. OKT3-HC and OKT3-LC domains originate from humanized variable regions of the heavy and light chains of OKT3. The Fc portion contains human IgG CH2 (C2) and CH3 (C3) domains. (**C**) Flow cytometric analysis of antigen binding using tri-31C2. Both the original construct (Ver. 7, green) and the optimized tri-31C2 variant (Ver. 18, magenta) bound to CD38-overexpressing CHO cell lines, as indicated by binding to isotype controls (dark blue). These data were previously presented at the American Society of Hematology Annual Meeting [18] and are shown here for illustrative purposes to explain antibody engineering concepts.

**Figure 3:**
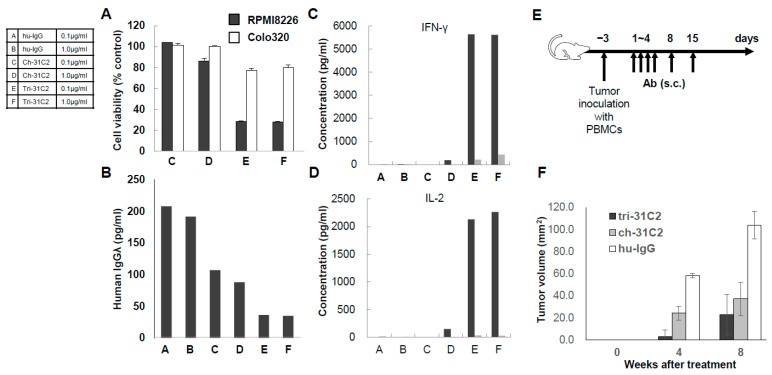
In vitro and in vivo antitumor activity of the tri-31C2 bispecific antibody. (**A**) In vitro tumor growth inhibition assay. CD38-positive RPMI 8226 cells or CD38-negative Colo320 cells (5000 cells/well) were cocultured with PBMCs at an E:T ratio of 5:1 in the presence of tri-31C2, ch-31C2, or a human IgG control. After 72 h, tumor growth was quantified by MTS assay. Tri-31C2 showed marked, PBMC-dependent growth inhibition compared with ch-31C2 and IgG controls. (**B**) Myeloma cell burden measured by human Igλ levels. Concentrations of secreted human Igλ (ELISA) from RPMI 8226 cultures confirmed enhanced tumor cell killing with tri-31C2. (**C**,**D**) Cytokine production. PBMC–myeloma cocultures treated with tri-31C2 for 24 h produced substantially higher levels of IFN-γ and IL-2 (measured by ELISA) relative to ch-31C2 or IgG, consistent with robust T-cell activation. (**E**,**F**) In vivo efficacy in a SCID mouse xenograft model. SCID mice were inoculated with RPMI 8226 cells and human PBMCs on day-3. Tri-31C2, ch-31C2, or control IgG (32 μg) was administered on days 1, 2, 3, 4, 8, and 15. Anti-asialo GM was given to deplete endogenous NK cells on days 0, 7, and 14. Tumor growth was monitored biweekly for up to 8 weeks. Tri-31C2 significantly suppressed tumor formation compared with control and ch-31C2. Error bars represent mean ± SD. These data were previously presented at the American Society of Hematology Annual Meeting [18] and are shown here for illustrative purposes to explain antibody engineering concepts.

To illustrate the principles of next-generation antibody engineering in multiple myeloma, we refer to a previously reported example of a T-cell–redirecting antibody targeting CD38 and CD3, designated tri-31C2 (Figure 2) [18]. This antibody was developed as a conceptual prototype to explore whether simultaneous engagement of tumor-associated antigens and CD3 on T cells could enhance anti-myeloma activity beyond that achieved with conventional monoclonal antibodies. Tri-31C2 was constructed by combining the variable regions of a chimeric anti-CD38 antibody and a humanized anti-CD3 antibody, fused to a human IgG1 Fc region to maintain structural stability and immunoglobulin-like pharmacokinetics. In previously presented studies, this design enabled efficient T-cell engagement and antigen-dependent cytotoxicity against CD38-positive myeloma cells, supporting the feasibility of CD3-redirecting strategies targeting plasma cell antigens (Figure 3). Importantly, the tri-31C2 example is presented here solely for illustrative purposes, highlighting the conceptual transition from conventional monoclonal antibodies toward multifunctional and T-cell–engaging antibody formats. These early observations anticipated the subsequent clinical success of CD38-directed bispecific antibodies and underscored the potential of rational antibody engineering to amplify antitumor immunity in multiple myeloma.

The functional characterization of tri-31C2, as disclosed in prior scientific presentations, demonstrated enhanced T-cell–mediated cytotoxicity in antigen-dependent coculture systems and xenograft models when compared with a conventional anti-CD38 monoclonal antibody backbone. In the context of this review, the tri-31C2 data are not presented as new experimental results but rather as a representative example illustrating how early antibody engineering efforts informed the development of modern T-cell–redirecting immunotherapies.

#### 2.2.2. Anti-SLAMF7 Antibody

Elotuzumab targets the signaling lymphocytic activation molecule family member 7 (SLAMF7, also known as CS1), which is encoded on chromosome 1q and is highly expressed in both plasma and NK cells. Its therapeutic efficacy is mainly derived from NK cell activation via SLAMF7–EAT-2 signaling and ADCC against SLAMF7^+^ myeloma cells.

Our recent molecular analyses have revealed that the soluble form of SLAMF7 (sSLAMF7) is not generated by proteolytic shedding but rather by alternative splicing of the SLAMF7 gene, yielding a unique variant X transcript that lacks the membrane-anchoring exon while preserving both IgV and IgC2 domains [19]. This variant X encodes a secreted protein that forms homotypic interactions with membrane SLAMF7 and promotes the proliferation of SLAMF7^+^ myeloma cells. Its expression level in tumor cells strongly correlates with serum sSLAMF7 concentration, which increases with disease progression. Importantly, elotuzumab neutralizes sSLAMF7, thereby inhibiting its growth-promoting effects.

Beyond direct tumor promotion, sSLAMF7 activates SLAMF7^+^ macrophages and fosters T-cell exhaustion through SLAMF7-mediated cross-talk, contributing to the immunosuppressive microenvironment of refractory myeloma. sSLAMF7 neutralization by elotuzumab reverses these effects, reducing the abundance of SLAMF7^+^ regulatory and exhausted T cells, and improving the immune landscape in relapsed/refractory MM. Therefore, high sSLAMF7 or variant X expression may serve as both biomarkers of disease progression and predictive markers of elotuzumab responsiveness, particularly in patients with 1q gain, in which SLAMF7 is upregulated.

Collectively, these findings redefine SLAMF7 as both an immune cell activator and a dual-function antigen in myeloma pathobiology, with its membrane and soluble forms exerting opposing effects that are therapeutically targetable by elotuzumab.

#### 2.2.3. B-Cell Maturation Antigen (BCMA)-Directed Antibodies and Bispecific Constructs

The BCMA is expressed almost exclusively in plasma cells and is a validated target in relapsed/refractory multiple myeloma (RRMM). Therapeutic modalities include ADCs, BsAbs, and CAR-T cells.

Among the BsAbs, teclistamab and elranatamab redirect T cells to lyse BCMA-positive myeloma cells via CD3 engagement. Contrastingly, belantamab mafodotin is an ADC that delivers a cytotoxic payload through monomethyl auristatin F.

Our recent structure-based study using AlphaFold 3 demonstrated the critical differences in the binding modes of anti-BCMA antibodies to membrane-bound and soluble BCMA. Mutations in the R27–P34 region of BCMA disrupt hydrogen bonding and promote antigen escape, particularly in the R27P and P34del variants [20]. Furthermore, soluble BCMA (sBCMA), generated by γ-secretase cleavage, exhibits a conformational shift that weakens hydrogen bonds with teclistamab but not with elranatamab or belantamab. Nevertheless, functional assays revealed that teclistamab maintains binding and cytotoxicity even in the presence of sBCMA, suggesting greater resilience to the “sBCMA sink” effect compared to elranatamab or belantamab.

These findings clarify the efficacy of teclistamab in patients previously exposed to BCMA-targeted therapy and provide a structural rationale for the differential antigen escape among BCMA-directed agents.

Collectively, these mechanistic advances highlight the diversity of antibody platforms in MM.

(1) CD38 antibodies modulate both immune and intrinsic tumor pathways (FOXM1-driven survival).
(2) SLAMF7 antibodies act via NK cell activation and immune synapse enhancement.
(3) BCMA antibodies and bispecifics employ structure-optimized binding to overcome antigen escape and soluble antigen interference.

Together, these strategies exemplify how rational antibody engineering and structural prediction are reshaping the treatment of refractory multiple myeloma and expanding the paradigm of precision immunotherapy.

#### 2.2.4. GPRC5D-Directed Antibodies

GPRC5D is an orphan G-protein-coupled receptor highly expressed on malignant plasma cells but minimally detected in normal tissues, except keratinized structures, making it an attractive therapeutic target distinct from BCMA. Talquetamab was the first GPRC5D × CD3 BsAbs to enter clinical practice and has broadened the immunotherapeutic landscape for RRMM.

##### Talquetamab Monotherapy

Preclinical studies have identified GPRC5D as a promising non-BCMA immunotherapeutic target. GPRC5D is highly expressed in malignant plasma cells, and the GPRC5D × CD3 bispecific antibody, JNJ-64407564, demonstrated potent antigen-specific T-cell–mediated cytotoxicity against myeloma cells in vitro and induced tumor regression in xenograft models [21].

In clinical phase 1–2 studies, talquetamab exhibited strong and durable responses in heavily pretreated RRMM, including patients previously exposed to BCMA-directed therapies. Overall response rates reached 74%, with ≥very good partial response rates of 59%, and meaningful activity was preserved following prior BCMA CAR-T or BCMA bispecific treatment. Class-specific toxicities, including dysgeusia, skin and nail changes, and cytokine release syndrome (CRS), are generally manageable. Collectively, these findings support GPRC5D as an independent immunotherapeutic axis that enables sequential targeting of plasma cell antigens beyond BCMA [22].

##### Dual Targeting of GPRC5D and BCMA

A recent major development is a dual-antigen targeting strategy that combines GPRC5D- and BCMA-directed BsAbs. The phase 1–2 trial demonstrated that talquetamab plus teclistamab achieved an overall response rate and a complete response rate of 80% and 52%, respectively, in heavily pretreated RRMM, with manageable CRS and infection rates and a delayed, attenuated pattern of keratin-related toxicities [23]. These results provide proof-of-concept that synergistic dual bispecific therapy targeting non-overlapping antigens may overcome antigen escape and broaden the durability of immunotherapy responses.

##### Clinical Integration

Talquetamab extends the antibody repertoire for RRMM by offering an antigen pathway independent of BCMA and CD38, robust efficacy after BCMA-directed therapy failure, compatibility with dual bispecific approaches (GPRC5D + BCMA), and opportunities for sequential or combination immune targeting. Together with the CD38-, SLAMF7-, and BCMA-focused platforms, talquetamab contributes to the emerging paradigm of multi-antigen, multi-platform immunotherapy in multiple myeloma, enabling more personalized and strategically layered treatment approaches.

### 2.3. T/NK-Cell and Myeloid Malignancies

Although antibody therapy in T-cell malignancies remains challenging owing to shared antigen expression, emerging targets such as CD30, CCR4, and CD123 have shown promise. Brentuximab vedotin (anti-CD30 ADC) and mogamulizumab (anti-CCR4) have established proof of concept for selective targeting in this difficult category.

#### 2.3.1. Antibody–Drug Conjugates and Targeted Antibodies in T/NK Malignancies

Brentuximab vedotin, an anti-CD30 ADC, has changed the treatment of classical Hodgkin’s lymphoma and systemic anaplastic large cell lymphoma. The AETHERA and ECHELON-2 trials demonstrated their ability to improve progression-free and overall survival when incorporated into frontline or consolidation therapy [24]. Its success validated the ADC platform for T-cell lymphomas, a historically difficult-to-treat group [24].

Mogamulizumab, a defucosylated anti-CCR4 antibody developed in Japan, is crucial in adult T-cell leukaemia/lymphoma (ATLL) [25], cutaneous T-cell lymphomas, and peripheral T cell lymphomas [26]. Its enhanced ADCC activity promotes CCR4+ malignant cell selective depletion, and remains among the few effective leukemic ATLL systemic therapies [25].

Tagraxofusp, a CD123-targeted IL-3 fusion toxin, was the first agent approved for blastic plasmacytoid dendritic cell neoplasm treatment, which is a rare and aggressive malignancy. Although its use is limited, tagraxofusp has demonstrated the feasibility of receptor-directed immunotoxins for the treatment of myeloid neoplasms [27].

#### 2.3.2. Antibody-Based Therapies in Acute Myeloid Leukemia

Gemtuzumab ozogamicin (GO) is a CD33-directed ADC that delivers the cytotoxic agent calicheamicin to leukemic blasts. Initially approved for relapsed acute myeloid leukemia (AML), GO was temporarily withdrawn due to safety concerns but was subsequently reapproved after evidence from the ALFA-0701 trial demonstrated that fractionated low-dose administration improved both efficacy and safety. Importantly, GO provides clinical benefits mainly in favorable and intermediate-risk AML, often as part of induction chemotherapy. Conversely, its activity in relapsed disease and the risk of hepatic veno-occlusive disease require careful patient selection. Although its role is more limited than that of modern T-cell–redirecting therapies and targeted inhibitors, GO remains a critical example of ADC development in myeloid malignancies.

### 2.4. Genomic Profiling and Molecular Stratification in Antibody-Based Hematologic Therapy

Genomic profiling expansion in hematologic malignancies has greatly enhanced diagnostic precision, risk stratification, and disease biology understanding. In Japan, the recent insurance approval and nationwide implementation of the HemeSight test marks a major milestone in genomic medicine. HemeSight enables comprehensive detection of single-nucleotide variants, indels, fusion genes, and structural abnormalities across 452 genes via integrated DNA/RNA sequencing of blood or bone marrow samples, with matched normal controls. Although the HemeSight panel has not yet been reported in a peer-reviewed publication, its clinical utility has been demonstrated in a prospective evaluation performed by the National Cancer Center Research Institute, which identified guideline-level actionable abnormalities in 85% of the tested cases.

Nevertheless, the direct link between genomic alterations and the clinical efficacy of antibody-based therapies remains limited. Current therapeutic monoclonal antibodies, including anti-CD20, anti-CD38, anti-SLAMF7, and BCMA-directed agents, are primarily selected based on cell-surface antigen expression and immunophenotyping rather than specific somatic mutations. Gene panel tests are not routinely used to predict therapeutic responses or resistance to antibody therapies. Nonetheless, comprehensive genomic profiling provides a critical foundation for precise immunotherapies. As sequencing approaches evolve toward whole-genome-, whole-exome-, and transcriptome-level analyses, integrative molecular signatures, including antigen expression programs, immune microenvironmental features, clonal architecture, and transcriptional states, are expected to aid in the prediction of antigen escape or downregulation, identification of patients most likely to benefit from bispecific antibodies or CAR-T cell therapy, discovery of resistance pathways associated with chronic antibody exposure, and optimization of rational combination therapies. Although genomic profiling has not yet been directly used to tailor antibody selection, it represents an essential diagnostic and biological platform that will increasingly support precision-guided antibody engineering and immunotherapy strategy development for hematological malignancies.

## 3. Antibody Therapeutics in Non-Malignant Hematologic Disorders

Further, antibody-based therapies have transformed nonmalignant hematologic disease treatment, including complement-mediated hemolytic disorders, coagulation factor deficiencies, and immune cytopenia. A diverse group of modalities, such as C5 inhibitors, FVIII-mimetic bispecific antibodies, FcRn inhibitors, and selective B-cell–depleting antibodies, is now approved across major regulatory regions. Table 2 summarizes the currently approved antibody therapeutics for non-malignant hematological conditions and their primary indications.

### 3.1. Complement Inhibition

Complement inhibition is a landmark in antibody therapeutics [28]. Eculizumab, a humanized anti-C5 antibody, is the first drug to prevent complement-mediated intravascular hemolysis in paroxysmal nocturnal hemoglobinuria (PNH) and atypical hemolytic uremic syndrome (aHUS). Ravulizumab, engineered to have an extended half-life, reduced the infusion frequency from biweekly to every 8 weeks.

Recently, Crovalimab (SKY59), developed using Sequential Monoclonal Antibody Recycling Technology (SMART-Ig), was approved in the USA, EU, Japan, and China for PNH treatment. Crovalimab employs pH-dependent binding and FcRn-mediated recycling, allowing repeated antigen engagement and an extended duration of complement blockade [29]. Unlike eculizumab and ravulizumab, crovalimab binds a distinct β-chain epitope and remains active in patients with the C5 p.Arg885His polymorphism resistant to eculizumab. The global phase 3 COMMODORE 2 and COMMODORE 3 trials demonstrated crovalimab’s non-inferiority to eculizumab in hemolysis control and transfusion avoidance, with comparable safety profiles [30]. A pooled safety analysis across COMMODORE 1–3 revealed no meningococcal infections and a lower serious infection rate (8.9 vs. 13.7 per 100 patient-years) compared with eculizumab [31]. Therefore, crovalimab represents a next-generation C5 inhibitor that combines efficacy, safety, and patient convenience via monthly subcutaneous self-administration.

### 3.2. Coagulation Disorders

Emicizumab, the first non-factor replacement therapy for hemophilia A, acts as a bispecific FVIIIa-mimetic antibody that bridges activated FIX (FIXa) and FX.

Developed by Chugai and Roche, the two Fab arms of emicizumab simultaneously bind to FIXa and FX to restore tenase complex formation, effectively replicating FVIIIa’s cofactor function [32]. The binding affinities (KD ≈ 1–2 µM) allow reversible catalytic cycling, ensuring physiological coagulation without excessive thrombin generation. Clinically, emicizumab provides sustained prophylaxis via subcutaneous injection every 1–4 weeks and is effective in patients treated with FVIII inhibitors. Long-term results from the HAVEN 1–4 trials demonstrated an annualized bleeding rate of 1.4 (95% CI, 1.1–1.7), with 82% of patients experiencing zero treated bleeds at 144 weeks [33].

Severe thrombotic or TMA events are rare and limited to cases with concomitant high-dose activated prothrombin complex concentrates.

Thus, emicizumab exemplifies the successful application of antibody engineering to functionally replace a missing coagulation protein, dramatically improving the outcomes and quality of life in patients with hemophilia A.

### 3.3. Immunodeficiency-Associated CD20-Positive B-Cell Lymphoproliferative Disorders

Immunodeficiency-associated CD20-positive B-cell lymphoproliferative disorders represent a distinct category of Epstein–Barr virus (EBV)-driven proliferation arising from impaired immune surveillance, including methotrexate-associated LPD, post-transplant LPD, EBV-positive mucocutaneous ulcers, and other EBV-positive B-cell proliferations. These disorders span a continuum from reactive polyclonal expansion to aggressive B-cell lymphomas with partial reversibility upon immune restoration [34]. Notably, rituximab is central to EBV-infected CD20 + B cell management, often producing a rapid response. Although rituximab is only formally approved for this indication in Japan, it is widely used off-label worldwide [35,36]. Their biology highlights the critical interplay between EBV infection, immune suppression, and B-cell expansion, positioning these disorders at the interface between benign and malignant hematological diseases.

### 3.4. FcRn Inhibition in Immune Thrombocytopenia

Emerging antibody therapies, such as anti-FcRn antibodies for immune thrombocytopenia and autoimmune hemolytic anemia, are expanding the therapeutic horizons in benign hematology.

The neonatal Fc receptor (FcRn) regulates IgG homeostasis by protecting IgG from lysosomal degradation via pH-dependent recycling. Disruption of this pathway has emerged as an effective therapeutic strategy for IgG-mediated autoimmune diseases.

Efgartigimod alfa is an engineered human IgG1 Fc fragment with a markedly increased affinity for FcRn at physiological pH. By competitively blocking IgG recycling, efgartigimod accelerates the degradation of pathogenic IgG, leading to a rapid reduction in total IgG levels, including antiplatelet antibodies in immune thrombocytopenia (ITP) [37].

In a phase 3 trial (ADVANCE IV), efgartigimod significantly improved durable platelet responses and reduced bleeding events in patients with chronic ITP refractory to standard therapies [38], resulting in regulatory approval across the United States, European Union, and Japan. Beyond ITP, FcRn inhibition represents a platform technology with potential applicability to autoimmune hemolytic anemia and other IgG-mediated hematologic disorders. Its safety profile and mechanism of action provide a therapeutic complement to B-cell–directed strategies such as rituximab, underscoring the expanding diversity of antibody-based therapies in benign hematology.

A summary of the major mechanistic classes of therapeutic antibodies used in nonmalignant hematologic disorders, including complement inhibitors, coagulation-modifying antibodies, and FcRn inhibitors, is presented in Table 3.

## 4. Future Perspectives

The rapid evolution of therapeutic antibodies in hematology is entering a new phase characterized by multifunctional engineering, T-cell redirection, and structure-guided optimization. While monoclonal antibodies, antibody–drug conjugates (ADCs), and bispecific antibodies have dramatically improved outcomes across malignant and non-malignant hematologic disorders, several unresolved clinical challenges remain critical for future development.

One major challenge associated with next-generation bispecific antibodies is treatment-related toxicity, particularly cytokine release syndrome (CRS), immune effector cell–associated neurotoxicity syndrome (ICANS), and infectious complications. Sustained T-cell engagement can lead to prolonged immune activation, T-cell exhaustion, and hypogammaglobulinemia, thereby increasing susceptibility to viral, bacterial, and opportunistic infections. Optimizing dosing schedules, step-up strategies, and combination regimens, as well as improving patient selection, will be essential to balance efficacy and safety in real-world practice.

For antibody–drug conjugates, off-target toxicity and payload-related adverse events remain significant limitations. The clinical experience with belantamab mafodotin has highlighted corneal toxicity as a class-specific adverse effect, reflecting antigen expression in non-malignant tissues and intracellular payload release. Future ADC development will require refined linker technologies, optimized payload selection, and improved tumor-specific antigen targeting to enhance the therapeutic window while minimizing systemic toxicity.

Another emerging concern is antigen escape and resistance. Loss of target antigen expression, such as CD19 downregulation after anti-CD19 therapy, as well as amino acid mutations in BCMA or GPRC5D, can reduce antibody binding and compromise treatment efficacy. Recent structural studies have demonstrated that mutations within key epitope regions of BCMA can alter hydrogen bonding and promote immune escape, underscoring the importance of epitope-resilient antibody design. These findings provide a strong rationale for dual-targeting or multi-antigen strategies to prevent clonal selection and prolong response durability [23].

In this context, combinatorial approaches using bispecific antibodies targeting non-overlapping antigens have shown promising clinical activity. Phase 2 data with extended follow-up demonstrated that dual bispecific therapy with talquetamab (GPRC5D × CD3) and teclistamab (BCMA × CD3) achieved deep and durable responses in heavily pretreated multiple myeloma, including extramedullary disease, with a manageable safety profile [23]. Furthermore, recent phase 3 evidence indicates that bispecific antibody–based combinations may substantially reshape the treatment landscape. In December 2025, teclistamab combined with daratumumab demonstrated markedly improved progression-free survival and depth of response compared with established daratumumab-based regimens, with predominantly low-grade and manageable cytokine release syndrome, supporting earlier integration of off-the-shelf bispecific antibody combinations with appropriate infection prophylaxis and supportive care [39].

Beyond antibody engineering itself, advances in artificial intelligence–driven structural prediction, such as AlphaFold 3, are beginning to transform antibody development. These tools enable precise epitope mapping, prediction of antigen escape variants, and rational optimization of antibody–antigen interactions, facilitating the design of next-generation antibodies with improved resilience to mutation and soluble antigen interference.

Finally, integration of genomic and transcriptomic profiling into antibody-based precision medicine represents an important future direction. Although current gene panel testing is primarily used for diagnosis and classification rather than treatment selection, comprehensive molecular profiling may ultimately identify transcriptional programs, antigen expression patterns, and immune microenvironment features that predict response, toxicity, or resistance to antibody therapies. As these technologies mature, the convergence of genomic medicine, structural biology, and immunotherapy is expected to guide patient stratification and rational combination strategies.

Collectively, future antibody therapeutics in hematology will increasingly rely on multi-antigen targeting, toxicity-aware engineering, and structure-guided design, supported by genomic and immunologic insights. These advances are poised to further refine precision immunotherapy and expand the clinical impact of antibody-based treatments across both malignant and non-malignant hematologic disorders.

## 5. Conclusions

Therapeutic antibodies have profoundly transformed hematologic medicine, enabling the targeted manipulation of malignant and immune pathways with increasing precision. The trajectory from rituximab to glycoengineered CD20 antibodies, radioimmunoconjugates to highly potent CD20 × CD3 bispecifics, and CD38/SLAMF7/BCMA antibodies to structurally optimized bispecific platforms in multiple myeloma illustrates the remarkable breadth of innovation achieved over the past two decades. In benign hematology, the advent of FcRn inhibitors, recycling of C5 antibodies, and FVIII-mimetic bispecifics has further expanded the reach of antibody therapeutics beyond oncology, improving outcomes in ITP, PNH, aHUS, and hemophilia A.

Collectively, these developments highlight a new era in which molecular engineering, structural modeling, and immunological insights converge to shape next-generation antibody therapeutics. As multifunctional designs, AI-assisted antigen prediction, and combination strategies with cellular therapies continue to advance, antibody-based treatments will remain at the forefront of precision medicine for both malignant and non-malignant hematologic disorders.

## Figures and Tables

**Table 1:** Regulatory approval status of antibody therapeutics for hematologic malignancies.

Drug (Generic Name)	Brand Name	Target	Modality	FDA	EMA	PMDA	Primary Indications
Rituximab	Rituxan/MabThera	CD20	mAb	√	√	√	B-cell NHL, CLL
Obinutuzumab	Gazyva/Gazyvaro	CD20	mAb	√	√	√	CLL, FL
Ofatumumab	Arzerra	CD20	mAb	√ *	√	√	CLL (withdrawn for oncology use in US)
Ibritumomab tiuxetan	Zevalin	CD20	Radioimmunoconjugate	√ **	√	√	R/R FL
Mosunetuzumab	Lunsumio	CD20 × CD3	BsAb	√	√	√	R/R FL
Glofitamab	Columvi	CD20 × CD3	BsAb	√	√		R/R DLBCL
Epcoritamab	Epkinly/Tepkinly	CD20 × CD3	BsAb	√	√	√	R/R LBCL, FL
Blinatumomab	Blincyto	CD19 × CD3	BsAb	√	√	√	B-ALL (R/R, MRD+)
Inotuzumab ozogamicin	Besponsa	CD22	ADC	√	√	√	B-ALL
Daratumumab	Darzalex	CD38	mAb	√	√	√	MM
Isatuximab	Sarclisa	CD38	mAb	√	√	√	MM
Elotuzumab	Empliciti	SLAMF7	mAb	√	√	√	MM
Teclistamab	Tecvayli	BCMA × CD3	BsAb	√	√	√	RRMM
Elranatamab	Elrexfio	BCMA × CD3	BsAb	√	√	√	RRMM
Linvoseltamab	Lynozyfic	BCMA × CD3 BsAb	BsAb	√	√		RRMM
Talquetamab	Talvey	GPRC5D × CD3	BsAb	√	√	√	RRMM
Belantamab mafodotin	Blenrep	BCMA	ADC	√	√	√	RRMM
Mogamulizumab	Poteligeo	CCR4	mAb	√	√	√	ATLL, PTCL, CTCL
Brentuximab vedotin	Adcetris	CD30	ADC	√	√	√	HL, PTCL, CTCL
Tagraxofusp	Elzonris	CD123	fusion protein	√	√	√	BPDCN
Gemtuzumab ozogamicin	Mylotarg	CD33	ADC	√	√	√	AML

* Ofatumumab oncology indication withdrawn in the US; remains approved for CLL in EMA/PMDA. ** Although ibritumomab tiuxetan is still approved, clinical availability is limited in the USA. ADC, Antibody–drug conjugate; ALL, Acute lymphoblastic leukemia; AML, Acute myeloid leukemia; ATLL, adult T-cell leukemia/lymphoma; BCMA, B-cell maturation antigen; BPDCN, blastic plasmacytoid dendritic cell neoplasm; BsAb, Bispecific antibody; CLL, Chronic lymphocytic leukemia; CTCL, Cutaneous T-cell lymphoma; DLBCL, Diffuse large B-cell lymphoma; EMA, European Medicines Agency; FDA, United States Food and Drug Administration; FL, Follicular lymphoma; GPRC5D, G protein-coupled receptor class C group 5 member D; HL, Hodgkin lymphoma; LBCL, Large B-cell lymphoma; mAb, Monoclonal antibody; MM, Multiple myeloma; MRD, Measurable residual disease; NHL, Non-Hodgkin lymphoma; PMDA, Pharmaceuticals and Medical Devices Agency; PTCL, peripheral T-cell lymphoma; R/R, Relapsed or refractory; RRMM, Relapsed or refractory multiple myeloma.

**Table 2:** Regulatory approval status of antibody therapeutics for non-malignant hematologic diseases.

Drug (Generic Name)	Brand Name	Target/Mechanism	Modality	FDA	EMA	PMDA	Primary Indications
Eculizumab	Soliris	C5 inhibition	Complement inhibitor	√	√	√	PNH, aHUS
Ravulizumab	Ultomiris	C5 inhibition	Complement inhibitor	√	√	√	PNH, aHUS
Crovalimab	Crovalimab	C5 inhibition (recycling Ab)	Recycling mAb	√ *	√	√	PNH
Emicizumab	Hemlibra	FVIII-mimetic bridging (FIXa–FX)	Bispecific mimetic	√	√	√	Hemophilia A (with/without inhibitors)
Efgartigimod alfa	Vyvgart	FcRn blockade → IgG reduction	FcRn inhibitor	√ *	√	√	ITP
Rituximab	Rituxan/MabThera	CD20	mAb	Off-label *	Off-label *	√	ITP, AIHA, immunodeficiency-associated B-LPD †

* Rituximab is not formally approved for autoimmune cytopenias in the US/EU, but widely used off-label. † PMDA officially approves rituximab for CD20-positive lymphoproliferative disorders under immunosuppression.

**Table 3:** Mechanisms of Major Non-Malignant Hematologic Antibody Therapies.

Class	Drugs	Mechanism of Action
Complement Inhibitors	Eculizumab Ravulizumab Crovalimab	Binds to complement component C5 and prevent its cleavage into C5a and C5b, thereby inhibiting membrane attack complex (MAC) formation
Coagulation-modifying Antibodies	Emicizumab	Bispecific FVIII-mimetic antibody that bridges FIXa and FX to restore intrinsic tenase activity and enable physiological thrombin generation
FcRn Inhibitors	Efgartigimod alfa	Blocks the neonatal Fc receptor (FcRn), accelerates IgG degradation, and reduces pathogenic IgG levels

This table summarizes the principal classes of antibody therapeutics used in non-malignant hematologic disorders, highlighting their mechanisms of action. Complement inhibitors block C5 cleavage to suppress terminal complement activation in PNH. Emicizumab functions as a FVIII-mimetic bispecific antibody that restores FIXa–FX bridging in hemophilia A. FcRn blockade accelerates IgG degradation and reduces IgG-mediated autoimmunity in disorders such as immune thrombocytopenia. C5, Complement component 5; C5a, Complement component 5a; C5b, Complement component 5b; FcRn, Neonatal Fc receptor; FIXa, Activated factor IX; FX, Factor X; FVIII, Factor VIII; IgG, Immunoglobulin G; MAC, Membrane attack complex.

## Data Availability

Not applicable.

## Abbreviations

ADC	antibody–drug conjugate
ADCC	antibody-dependent cellular cytotoxicity
aHUS	atypical hemolytic uremic syndrome
ALL	acute lymphoblastic leukemia
AML	acute myeloid leukemia
ATLL	adult T-cell leukemia/lymphoma
BCMA	B-cell maturation antigen
BiTE	bispecific T-cell engager
BsAb	bispecific antibody
CAR-T	chimeric antigen receptor T cell
CDC	complement-dependent cytotoxicity
CLL	chronic lymphocytic leukemia
CR	complete response
CRS	cytokine release syndrome
DLBCL	diffuse large B-cell lymphoma
EBV	Epstein–Barr virus
FcRn	neonatal Fc receptor
FVIII	factor VIII
GPRC5D	G-protein–coupled receptor class C group 5 member D
IgG	immunoglobulin G
IMiD	immunomodulatory drug
ITP	immune thrombocytopenia
MHC	major histocompatibility complex
MM	multiple myeloma
MRD	minimal residual disease
NK cell	natural killer cell
ORR	overall response rate
PBMC	peripheral blood mononuclear cell
PFS	progression-free survival
PNH	paroxysmal nocturnal hemoglobinuria
R/R	relapsed or refractory
RRMM	relapsed or refractory multiple myeloma
ROS	reactive oxygen species
SLAMF7	signaling lymphocytic activation molecule family member 7
sBCMA	soluble B-cell maturation antigen
sSLAMF7	soluble SLAMF7
TMA	thrombotic microangiopathy
WHO	World Health Organization
